# Two sexes, one body: intra- and intersex covariation of gamete phenotypes in simultaneous hermaphrodites

**DOI:** 10.1002/ece3.1035

**Published:** 2014-03-18

**Authors:** Keyne Monro, Dustin J Marshall

**Affiliations:** School of Biological Sciences, Monash UniversityMelbourne, Victoria, 3800, Australia

**Keywords:** Broadcast spawners, egg traits, marine invertebrates, sexual conflict, sperm traits

## Abstract

By harboring male and female functions in the same genome and expressing them in every individual, simultaneous hermaphrodites may incur sexual conflict unless both sex functions can evolve phenotypic optima independently of each other. The first step toward understanding their capacity to do so lies in understanding whether sex functions are phenotypically correlated within individuals, but remarkably few data address this issue. We tested the potential for intra- and intersex covariation of gamete phenotypes to mediate sexual conflict in broadcast-spawning hermaphrodites (the ascidians *Ciona intestinalis* and *Pyura praeputialis*), for which sex-specific selection acts predominantly on sperm–egg interactions in the water column. In both species, gamete phenotypes covaried within and across sex functions, implying that selection may be unable to target them independently because its direct effects on male gametes translate into correlated effects on female gametes and *vice versa*. This alone does not preclude the evolution of a different phenotypic optimum for each sex function, but imposes the more restrictive requirement that selection – which ultimately sorts among whole individuals, not sex functions – aligns with the direction in which gamete phenotypes covary at this level.

## Introduction

Conflicting evolutionary interests of males and females over mating interactions can generate sexually antagonistic selection, with the potential to drive sexual arms races (adaptation and counter-adaptation in reproductive phenotypes) that can lead rapidly to reproductive isolation and speciation (Arnqvist and Rowe 2005; Chapman 2006; Parker 2006). Evolving sexual dimorphism, or sex-specific phenotypic optima, may ameliorate sexual conflict (Lande 1980; Bonduriansky and Chenoweth 2009), but what happens when such options are limited, as in hermaphrodite taxa that combine sexes (or sex functions) within individuals?

The lack of sexual dimorphism in simultaneous hermaphrodites (those expressing life-long hermaphroditism, our focus here unless otherwise stated) led Darwin (1874) to famously dismiss the role of sexual selection in their evolution. However, sexual selection arises ultimately from anisogamy (Parker et al. 1972; Schärer et al. 2012), which may have first evolved under a primitive form of sexual conflict (Parker 2006), and is certainly present in hermaphrodites. Even in sessile hermaphrodites (e.g., plants and marine invertebrates) with few opportunities to actively pursue mates or express sex roles behaviorally, sexual conflict has ample scope to manifest in primary sexual characteristics, such as the phenotypes of gametes themselves. It may even be more direct and intense in hermaphrodites than in separate-sexed species (gonochorists) because the expression of both male and female components of fitness by every individual means that no sex-related traits are masked from selection for one or more generations, as occurs in gonochorists (Michiels 1998; Bedhomme et al. 2009; Abbott 2011). Every generation, moreover, hermaphrodite individuals compete with all others in the population for their contribution to the next generation, whereas males compete only with males (and females with females) for this contribution in gonochorists (Charnov 1979; Leonard 2006). Hermaphrodites may therefore offer excellent systems for testing theories about sexual conflict, but progress is hampered by the lack of quantitative data on key traits underlying their mating interactions (Arnqvist and Rowe 2005; Schärer and Pen 2013). The vast majority of research into sexual conflict deals with gonochorists, and the exceptions involving animal hermaphrodites focus mainly on the copulatory behaviors of pairwise-mating taxa, such as flatworms, sea slugs, and snails (see, e.g., Koene and Schulenburg 2005; Anthes and Michiels 2007; Schärer and Janicke 2009 and references therein), that are a subset of the hermaphrodite mating systems seen in the animal kingdom (Jarne and Auld 2006).

The challenge for simultaneous hermaphrodites is to resolve sexually antagonistic selection while harboring male and female functions in the same genome and expressing them in every individual (Charnov 1979; Michiels 1998; Leonard 2006), which may predispose hermaphrodites to sexual conflict unless sex functions can evolve phenotypic optima independently of one other (Parker 2006). Ultimately, such capacity depends on the degree to which sex functions covary genetically, and whether they do so in the direction that is favored by selection upon each (Abbott 2011), but to our knowledge, this remains untested for any animal taxon. A key first step lies in understanding whether sex functions are phenotypically correlated within individuals (Morgan 1994; Michiels 1998), but to date, there are remarkably few data addressing this issue. Here, we test the potential for intra- and intersex covariation of gamete phenotypes to mediate sexual conflict in simultaneous hermaphrodites with the ancestral mating strategy of broadcast spawning (Wray 1995; Levitan 1996). While traits must vary among individuals to evolve, nonindependence of traits owing to functional or developmental integration can constrain evolution by translating direct selection on one trait into correlated effects on others (in effect, restricting the distribution of phenotypic variance available to selection), and by setting upper bounds on the underlying genetic variance that directs any evolutionary response (Lande and Arnold 1983; Morgan 1994; Blows 2007).

Our study species, *Ciona intestinalis* (Linnaeus, 1767) and *Pyura praeputialis* (Heller, 1878; both named by genus hereafter) are ascidians, the most primitive of living chordates (Lambert 2005). Nearly, all ascidians are simultaneous hermaphrodites and solitary forms, including *Ciona* and *Pyura*, spawn eggs, and sperm synchronously into the sea where fertilization and development occur. Egg size is under selection in both study species (Marshall et al. 2002; Marshall and Keough 2003b), and sperm morphology has demonstrable effects on fertilization success in other broadcast spawners (Fitzpatrick et al. 2012; Johnson et al. 2013), making the integration of primary sexual characteristics within hermaphrodite individuals a prime battleground for sexual conflict during mating.

## Material and methods

*Ciona* and *Pyura* are sessile as adults and live in aggregations on various submerged surfaces in coastal marine environments. Individuals are normally self-sterile (Lambert 2005) and at dawn or low tide, spawn sperm and eggs synchronously into the sea for cross-fertilization. There is little opportunity to choose or compete for mates beforehand (Evans and Sherman 2013). Fertilization is by no means assured and depends principally on rates of sperm–egg collision in the water column, which in turn depend on patterns of spawning that mediate gamete densities (e.g., the duration of gamete release and its timing relative to mates) plus the traits of gametes themselves (Marshall and Keough 2008; Lotterhos and Levitan 2010). For each species, individuals were sampled from a wild population in Port Phillip Bay (Victoria, Australia), fed for several days in darkened aquaria to prevent spawning, then dissected to harvest the accumulated gametes from the hermaphrodite gonads of 36 sexually mature specimens of *Ciona* and 29 such specimens of *Pyura*.

### Harvesting of gametes

*Ciona* has a gelatinous body encased in a thin translucent tunic. When the tunic is removed, sexually mature individuals have a visible testis and ovary, plus conspicuous gonoducts (a spermiduct and oviduct running dorsally along the intestine from the gonads to the exhalant siphon) where ripe gametes pool before spawning (Christiaen et al. 2009). To harvest gametes from mature individuals, we pierced their gonoducts and gently extracted gametes using a 1 ml syringe, keeping sperm and eggs isolated. An individual's eggs were dispensed into a dish of filtered seawater, covered, and refrigerated for 30 min before processing to allow the expansion of all accessory structures (see below; Berrill 1947; Levitan 1995; Marshall and Keough 2003a). An individual's sperm were processed without delay, but kept refrigerated at high concentration in the syringe until use to limit deterioration. Individuals were blotted to remove excess water, and their weight without tunics (which were fouled by epibiota) recorded as a measure of body size including all somatic and gonadal tissue, minus previously sampled gametes.

*Pyura* has a fleshy body encased in a tough opaque tunic. As this species does not accumulate gametes in gonoducts, we opened animals sagittally through their siphons, removed their tunics, and exposed the distinctive green ovaries and orange testes attached to the body walls of sexually mature individuals (Rius and Teske 2011). At this point, individuals were blotted and weighed without tunics to record body size including all somatic and gonadal tissue, plus subsequently sampled gametes (note that this minor difference in measures of body size between species was required by their differing anatomies, but is unlikely to affect our results because the sampled gametes contributed negligible weight to *Ciona* individuals and data from each species were ultimately analyzed separately). We then dissected the gonads of each *Pyura* into dishes, isolating testes and ovaries as much as possible, and gently pressed the tissue in a few drops of filtered seawater to liberate ripe gametes. We rinsed each suspension with filtered seawater through a 100-*μ*m filter that retained eggs while passing sperm into a small beaker below (Crean and Marshall 2008). Gametes were then treated as above for *Ciona* until processing.

### Measurement of gamete phenotypes

**Figure 1:**
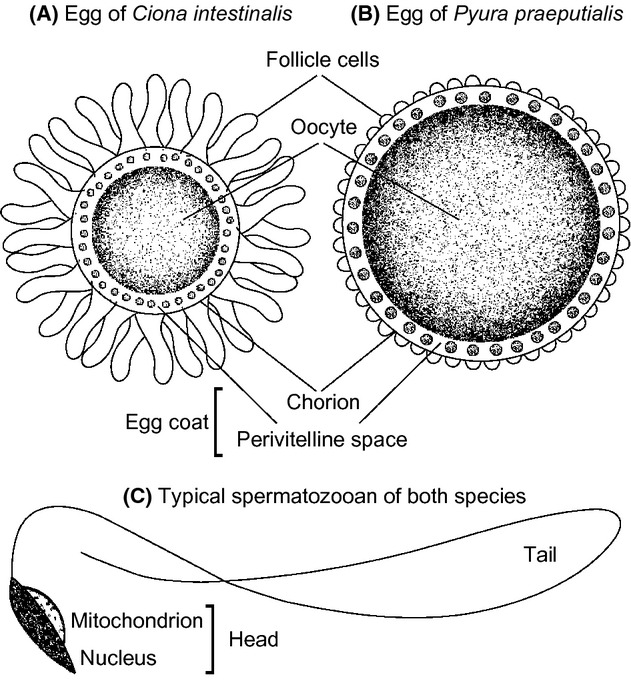
Focal traits measured on gametes in two species of simultaneous hermaphrodite (see Fig. 2 for scales).

The eggs of broadcast-spawning ascidians vary within and among species in two respects. First is in the quantity and quality of yolk in the oocyte, which is the entire maternal contribution to postzygotic performance (Marshall et al. 2002; Marshall and Keough 2003b). Second is in the accessory structures coating the oocyte, which have mainly prezygotic functions (e.g., increasing the effective target size for sperm, blocking self-fertilization and polyspermy, aiding species recognition; Lambert 2005; Podolsky 2004, 2001). In our species, these structures comprise a membranous chorion (in mammals, the zona pellucida) enclosing a layer of test cells in perivitelline fluid, plus a layer of follicle cells on the chorion surface (Fig. 1A and B). For each individual (the units of replication in our analytical design), we photographed 15 freshly harvested, healthy eggs (the sources of sampling error in our design) using a digital camera mounted to a compound microscope at 1000× magnification. We measured the cross-sectional areas of oocyte and egg coat (the chorion and perivitelline space) using the image-processing software, ImageJ (http://rsb.info.nih.gov/ij). For *Ciona*, we also measured the mean length of three follicle cells per egg, which are prominent in this species but only minor structures in *Pyura* (Fig. 1).

Compared with internal fertilizers, sperm of externally fertilizing ascidians are small and simple. Each spermatozoan comprises an awl-shaped nucleus carrying the paternal genome and a simple tail providing motility, but lack the mid-piece typical of other taxa. Instead, the cell is powered by a single mitochondrion (flanking the nucleus and forming with it the sperm head; Fig. 1C), which is shed during egg coat penetration and may provide the necessary force for this process (Lambert 1982; Franzén 1992). As for eggs, we photographed 15 freshly harvested spermatozoa per individual, measuring each cell's maximum head length, maximum head width, and tail length (Fig. 1C) in ImageJ.

### Statistical analyses

To test for intra- and intersex covariation in gamete phenotypes across individuals of each species, we analyzed gamete traits in multivariate linear mixed models implemented with restricted maximum likelihood in SAS 9.3 (SAS Institute Inc., Cary, NC). We first modeled the set of traits measured on both species (sperm head length, sperm head width, sperm tail length, egg oocyte area, and egg coat area), later adding mean follicle-cell length to a species-specific model for *Ciona*. Traits were corrected for individual body size by modeling weight as a covariate. We compared models using likelihood ratio tests (LRTs) based on a *χ*^2^ distribution with degrees of freedom as the number of parameters by which models differed.

The first model was



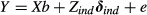
1

where ***Y*** is the vector of observations for gamete traits (scaled to a mean of 0 and standard deviation of 1 for each trait to express all traits in common units for multivariate analysis), ***X*** is the design matrix for the fixed effect of individual body size (***b***), and ***Z***_ind_ is the design matrix for the random effect of individual, regardless of species. The phenotypic variance of gamete traits across individuals (***δ***_ind_) was modeled as an unstructured covariance matrix, and the error term (***e***) was partitioned to predict the residual variance among gametes for each trait (which fit the data significantly better than modeling a pooled error term; LRT: *χ*^2^_(4) _= 71.6, *P *<* *0.01).

The second model was



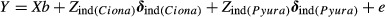
2

which differed to model 1 by predicting the phenotypic variance of gamete traits across individuals of *Ciona* and *Pyura* separately, as is more biologically realistic (note that our initial fitting of model 1 to pooled data served only as a null model against which to formally test for species-specific trait variation). Model 2 fit the data significantly better than model 1 (LRT: *χ*^2^_(15) _= 38.1, *P *<* *0.01), confirming that overall patterns of variation in gamete traits differed between species and leading us to fit a separate model 1 for each. At this point, the mean follicle-cell length of eggs was introduced as an additional trait in the *Ciona* model.

Next, we further partitioned each model's error term to predict the trait-specific residual variance among gametes for every individual. This added a substantial number of covariance parameters to both models, but our data were sufficient to do so since each trait was scored on 15 gametes at this level. The fit of both models was significantly improved by such partitioning (LRT for *Ciona*:*χ*^2^_(170) _= 417.5, *P *<* *0.01; LRT for *Pyura*:*χ*^2^_(140) _= 219.9, *P *<* *0.01), implying that the within-individual variability of gametes differed across individuals of each species. Last, we tested the significance of all individual-level covariance parameters (elements of ***δ***_ind_ for each species) using LRTs. Tests were one-tailed for variances, two-tailed for covariances, and corrected for multiplicity (Benjamini and Hochberg 1995; Fry 2004).

## Results

**Figure 2:**
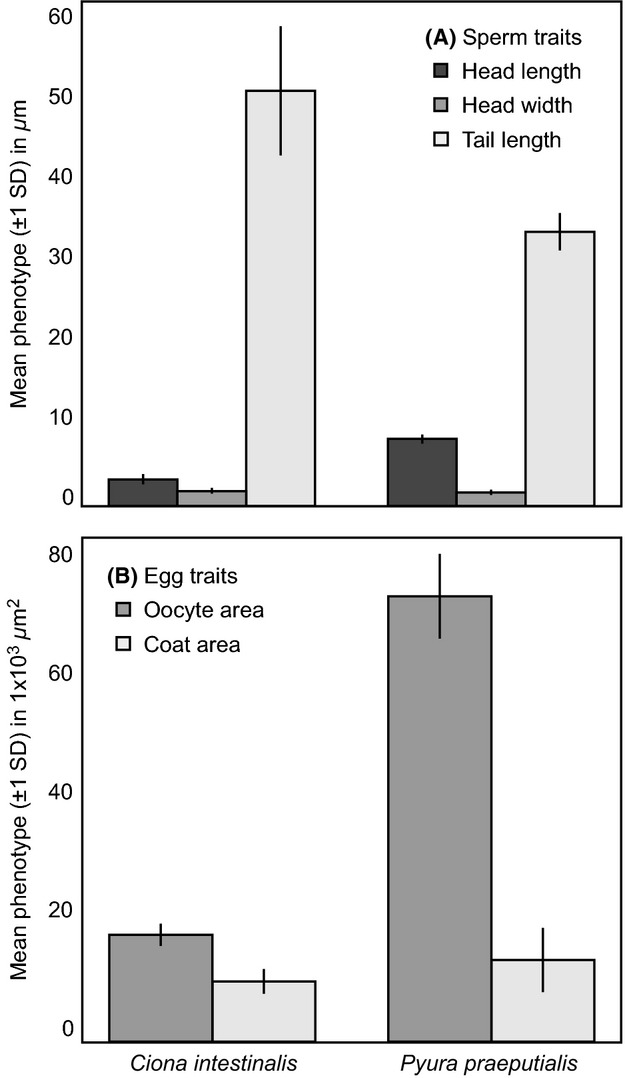
Mean phenotypes of gametes in two species of simultaneous hermaphrodite (mean follicle-cell length of *Ciona* eggs = 71.69 *μ*m ± 12.28 SD).

On average relative to *Ciona*,*Pyura* produced sperm with longer bodies and shorter tails (Fig. 2A), and eggs with greater area of oocyte proportional to coat (Fig. 2B). In both species, gamete phenotypes varied significantly across individuals: We not only detected significant variation in all focal traits per sex function, but we also detected significant covariation within and across them that was often, although not always, species-specific and often involved the same traits (Table 1).

**Table 1:** The phenotypic variance of gametes across hermaphrodite individuals. White blocks present the variance of each focal trait per sex, plus intrasex covariances and correlations (italicized). Gray blocks present intersex covariances and correlations (italicized). Predicted values are followed by standard errors and are in bold when significant (*P *<* *0.05).

	Sperm traits			Egg traits		
Head length (*μ*m)	Head width (*μ*m)	Tail length (*μ*m)	Oocyte area (*μ*m^2^)	Coat area (*μ*m^2^)	FC length (*μ*m)
(a) *Ciona intestinalis*						
Sperm traits						
Head length	**0.29 ± 0.08**	*0.33 ***±*** 0.22*	***0.69 *****±***** 0.13***	*−0.01 ***±*** 0.19*	*0.08 ***±*** 0.19*	*0.16 ***±*** 0.19*
Head width	0.06 **±** 0.04	**0.11 ± 0.05**	***0.50 *****±***** 0.21***	*0.12 *±* 0.24*	***0.50 *****±***** 0.19***	*0.42 ***±*** 0.20*
Tail length	**0.18 ± 0.06**	**0.08 ± 0.04**	**0.23 ± 0.07**	*0.28 *±* 0.19*	***0.50 *****±***** 0.15***	*0.16 ***±*** 0.19*
Egg traits						
Oocyte area	0.00 **±** 0.08	0.03 ± 0.06	0.10 ± 0.07	**0.52 ± 0.14**	*0.02 ***±*** 0.18*	*0.23 ***±*** 0.18*
Coat area	0.03 **±** 0.08	**0.13 ± 0.06**	**0.18 ± 0.08**	0.01 **±** 0.10	**0.59 ± 0.15**	0.00 **±** *0.18*
FC length	0.07 **±** 0.09	0.11 **±** 0.06	0.06 **±** 0.08	0.14 **±** 0.11	0.00 **±** 0.11	**0.67 ± 0.17**
(b) *Pyura praeputialis*						
Sperm traits						
Head length	**0.16 ± 0.06**	*−0.21 ***±*** 0.25*	*0.09 ***±*** 0.26*	*0.10 ***±*** 0.24*	*−0.08 ***±*** 0.26*	
Head width	*−*0.03 **±** 0.04	**0.18 ± 0.06**	***0.51 *****±***** 0.21***	*0.20 ***±*** 0.24*	*−**0.49 *****±***** 0.21***	
Tail length	0.02 **±** 0.04	**0.09 ± 0.05**	**0.17 ± 0.07**	*0.19 ***±*** 0.24*	*−**0.62 *****±***** 0.18***	
Egg traits						
Oocyte area	0.02 **±** 0.05	0.05 ± 0.06	0.04 **±** 0.06	**0.28 ± 0.09**	*−**0.59 *****±***** 0.18***	
Coat area	*−*0.02 **±** 0.05	*−***0.09 ± 0.05**	*−***0.12 ± 0.05**	*−***0.14 ± 0.06**	**0.20 ± 0.07**	

FC, follicle cell.

Within sex functions of *Ciona*, both the length and width of the sperm head covaried positively with tail length, but were themselves uncorrelated. Thus, sperm with longer tails had longer or wider bodies, but were not necessarily larger overall. No covariation between egg traits was detected. Across sex functions, sperm head width and tail length covaried positively with the area of egg coat, such that individuals producing longer, wider sperm also produced thicker-coated eggs (Table 1a).

Within sex functions of *Pyura*, sperm head width and tail length again covaried positively (as in *Ciona*), whereas the areas of oocyte and egg coat covaried negatively, implying that eggs with larger oocytes had thinner coats and *vice versa*. Across sex functions, sperm head width and tail length covaried negatively with the area of egg coat (contrasting with the signs of their covariances in *Ciona*), such that individuals producing longer, wider sperm also produced thinner-coated eggs (Table 1b).

## Discussion

We demonstrate intra- and intersex covariation of gamete phenotypes in *Ciona* and *Pyura*, two broadcast-spawning hermaphrodites in which sex-specific selection acts predominantly on sperm–egg interactions in the water column (Lotterhos and Levitan 2010; Evans and Sherman 2013). Thus, despite abundant variation in gamete phenotypes, selection may be unable to target sex functions independently because their covariation translates direct selection on sperm phenotype into correlated effects on egg phenotype and *vice versa* (Lande and Arnold 1983; Morgan 1994). This alone does not preclude the evolution of a different phenotypic optimum for each sex function, but imposes the more restrictive requirement that selection – which ultimately sorts among whole individuals, not sex functions – aligns with the direction in which gamete phenotypes covary at this level (Blows 2007).

Such covariation could be an intrinsic property of hermaphroditism – for example, because an individual's sex functions share a genetic basis, or because each individual must allocate a finite level of resources between these functions (Charnov 1979; Bedhomme et al. 2009). Alternatively, it could be shaped extrinsically by the selection pressures operating in the mating environment, including selection exerted by the gametes of other individuals. For our study species, mating environments range from sperm-saturated to sperm-limited, exposing both sex functions to selection for increased fertilization success (Crean and Marshall 2008; Levitan 2008). For sperm, too few data currently exist to generalize about what constitutes a successful phenotype (Evans and Sherman 2013), but head–tail covariation in sperm of both *Ciona* and *Pyura* suggests that selection may target them as functionally integrated units, as has been demonstrated in several broadcast-spawning gonochorists (Fitzpatrick et al. 2012; Johnson et al. 2013). For eggs, fertilization success often covaries with the effective target size they present to sperm (Levitan 1996; Marshall and Keough 2003b), which increases the probability of sperm–egg collision in the water column and is maximized with the least energetic cost by expanding the accessory structures of the egg coat (Podolsky 2001, 2004). Unlike the oocyte, these structures are assumed to be independent of postzygotic performance and evolve more strictly in response to mating interactions (Levitan 1996; Podolsky 2004) – an assumption that is supported here for *Ciona*, in which the areas of egg coat and oocyte were decoupled, but not for *Pyura*, in which their covariation links the pre- and postzygotic selection pressures acting on eggs.

Hermaphroditism aside, theory and data suggest that the evolutionary interests of the sexes in broadcast spawners may well be in conflict (because sperm are selected to secure fertilizations rapidly, whereas eggs are selected to slow fertilization rates in order to avoid polyspermy) over the continuum of sperm availability in the water column (Bode and Marshall 2007; Levitan 2008). The added problem unique to hermaphrodites is that synchronous release of sperm and eggs exposes both of an individual's sex functions to the same mating environment simultaneously and forces it to compete for fertilizations with all other individuals in the population including potential mates (Leonard 2006; Abbott 2011). Thus, our key result, the detection in both our study organisms of covariation between sperm morphology and the egg coat with which sperm must interact before penetrating the oocyte, may have important implications for sexual conflict in hermaphrodites. It raises the possibility of conflicting sex-specific selection on gamete phenotypes within an individual's lifetime and requires such selection to be accommodated or resolved intra-individually. Intriguingly, although *Ciona* and *Pyura* belong to evolutionarily divergent groups within the ascidians, intersex covariation involved the same gamete traits in both species but changed direction between them. It would be interesting to know whether this is mere coincidence or a signal that these particular traits play a functional role in the evolution of reproductive isolation in such mating systems.

In conclusion, our study offers a novel perspective on the potential for gamete phenotypes to be a source of sexual conflict during mating and fertilization in simultaneous hermaphrodites. Understanding whether such potential translates into actual conflict (and how hermaphrodites might resolve this) requires a better understanding of the extent to which these phenotypes are intrinsically (e.g., genetically) versus extrinsically (e.g., environmentally) controlled, the specific selection pressures acting on them and whether such pressures act against gamete covariation at the genetic level, than currently exists for any animal taxon. Doing so, however, will not only help to clarify the taxonomic range of sexual conflict, but can provide a more unified view of this phenomenon by highlighting emergent properties in how it arises, manifests and is resolved in different mating systems.

## References

[b1] Abbott JK (2011). Intra-locus sexual conflict and sexually antagonistic genetic variation in hermaphroditic animals. Proceed. Royal Soc. B: Biol. Sci.

[b2] Anthes N, Michiels NK (2007). Precopulatory stabbing, hypodermic injections and unilateral copulations in a hermaphroditic sea slug. Biol. Lett.

[b3] Arnqvist G, Rowe L (2005). Sexual conflict.

[b4] Bedhomme S, Bernasconi G, Koene JM, Lankinen Å, Arathi HS, Michiels NK (2009). How does breeding system variation modulate sexual antagonism?. Biol. Lett.

[b5] Benjamini Y, Hochberg Y (1995). Controlling the false discovery rate: a practical and powerful approach to multiple testing. J. Royal Stat. Soc. Series B.

[b6] Berrill NJ (1947). The development and growth of *Ciona*. J. Marine Biol. Assoc. U.K.

[b7] Blows MW (2007). A tale of two matrices: multivariate approaches in evolutionary biology. J. Evol. Biol.

[b8] Bode M, Marshall DJ (2007). The quick and the dead? Sperm competition and sexual conflict in the sea. Evolution.

[b9] Bonduriansky R, Chenoweth SF (2009). Intralocus sexual conflict. Trends Ecol. Evol.

[b10] Chapman T (2006). Evolutionary conflicts of interest between males and females. Curr. Biol.

[b11] Charnov EL (1979). Simultaneous hermaphroditism and sexual selection. Proc. Natl. Acad. Sci. U.S.A.

[b12] Christiaen L, Wagner E, Shi W, Levine M (2009). The sea squirt *Ciona intestinalis*. Cold Spr. Harb. Prot.

[b13] Crean AJ, Marshall DJ (2008). Gamete plasticity in a broadcast spawning marine invertebrate. Proc. Natl. Acad. Sci. U.S.A.

[b14] Darwin C (1874). The descent of man, and selection in relation to sex.

[b15] Evans JP, Sherman CDH (2013). Sexual selection and the evolution of egg-sperm interactions in broadcast-spawning invertebrates. Biol. Bull.

[b16] Fitzpatrick JL, Simmons LW, Evans JP (2012). Complex patterns of multivariate selection on the ejaculate of a broadcast spawning marine invertebrate. Evolution.

[b17] Franzén Å (1992). Spermatozoan ultrastructure and spermatogenesis in aplousobranch ascidians, with some phylogenetic considerations. Mar. Biol.

[b18] Fry JD, Saxton AM (2004). Estimation of genetic variances and covariances by restricted maximum likelihood using PROC MIXED. Genetic analysis of complex traits using SAS.

[b19] Jarne P, Auld JR (2006). Animals mix it up too: the distribution of self-fertilization among hermaphroditic animals. Evolution.

[b20] Johnson DW, Monro K, Marshall DJ (2013). The maintenance of sperm variability: context-dependent selection on sperm morphology in a broadcast spawning invertebrate. Evolution.

[b21] Koene J, Schulenburg H (2005). Shooting darts: co-evolution and counter-adaptation in hermaphroditic snails. BMC Evol. Biol.

[b22] Lambert CC (1982). The ascidian sperm reaction. Am. Zool.

[b23] Lambert CC (2005). Historical introduction, overview, and reproductive biology of the protochordates. Can. J. Zool.

[b24] Lande R (1980). Sexual dimorphism, sexual selection, and adaptation in polygenic characters. Evolution.

[b25] Lande R, Arnold SJ (1983). The measurement of selection on correlated characters. Evolution.

[b26] Leonard JL (2006). Sexual selection: lessons from hermaphrodite mating systems. Integr. Comp. Biol.

[b27] Levitan DR, McEdward LR (1995). The ecology of fertilization in free-spawning invertebrates. Ecology of marine invertebrate larvae.

[b28] Levitan DR (1996). Effects of gamete traits on fertilization in the sea and the evolution of sexual dimorphism. Nature.

[b29] Levitan DR (2008). Gamete traits influence the variance in reproductive success, the intensity of sexual selection, and the outcome of sexual conflict among congeneric sea urchins. Evolution.

[b30] Lotterhos KE, Levitan DR, Leonard JL, Córdoba-Aguilar A (2010). Gamete release and spawning behavior in broadcast spawning marine invertebrates. The evolution of primary sexual characters in animals.

[b31] Marshall DJ, Keough MJ (2003a). Effects of settler size and density on early postsettlement survival of *Ciona intestinalis* in the field. Mar. Ecol. Prog. Ser.

[b32] Marshall DJ, Keough MJ (2003b). Sources of variation in larval quality for free-spawning marine invertebrates: egg size and the local sperm environment. Invert. Rep. Dev.

[b33] Marshall DJ, Keough MJ (2008). The evolutionary ecology of offspring size in marine invertebrates. Adv. Mar. Biol.

[b34] Marshall DJ, Styan CA, Keough MJ (2002). Sperm environment affects offspring quality in broadcast spawning marine invertebrates. Ecol. Lett.

[b35] Michiels NK, Birkhead TR, Møller AP (1998). Mating conflicts and sperm competition in simultaneous hermaphrodites. Sperm competition and sexual selection.

[b36] Morgan MT (1994). Models of sexual selection in hermaphrodites, especially plants. Am. Nat.

[b37] Parker GA (2006). Sexual conflict over mating and fertilization: an overview. Philos. Trans. Royal Soc. B: Biol. Sci.

[b38] Parker GA, Baker RR, Smith VGF (1972). The origin and evolution of gamete dimorphism and the male-female phenomenon. J. Theor. Biol.

[b39] Podolsky RD (2001). Evolution of egg target size: an analysis of selection on correlated characters. Evolution.

[b40] Podolsky RD (2004). Life-history consequences of investment in free-spawned eggs and their accessory coats. Am. Nat.

[b41] Rius M, Teske PR (2011). A revision of the *Pyura stolonifera* species complex (Tunicata, Ascidiacea), with a description of a new species from Australia. Zootaxa.

[b42] Schärer L, Janicke T (2009). Sex allocation and sexual conflict in simultaneously hermaphroditic animals. Biol. Lett.

[b43] Schärer L, Pen I (2013). Sex allocation and investment into pre- and post-copulatory traits in simultaneous hermaphrodites: the role of polyandry and local sperm competition. Philos. Trans. Royal Soc. B: Biol. Sci.

[b44] Schärer L, Rowe L, Arnqvist G (2012). Anisogamy, chance and the evolution of sex roles. Trends Ecol. Evol.

[b45] Wray G, McEdward LR (1995). Evolution of larvae and developmental modes. Ecology of marine invertebrate larvae.

